# Crimean-Congo Hemorrhagic Fever Virus-Infected Hepatocytes Induce ER-Stress and Apoptosis Crosstalk

**DOI:** 10.1371/journal.pone.0029712

**Published:** 2012-01-06

**Authors:** Raquel Rodrigues, Gláucia Paranhos-Baccalà, Guy Vernet, Christophe N. Peyrefitte

**Affiliations:** 1 Emerging Pathogens Laboratory, Fondation Mérieux, Lyon, France; 2 Unité de Virologie, Institut de Recherche Biomédicale des Armées, La Tronche, France; National Institute for Infectious Diseases (L. Spallanzani), Italy

## Abstract

Crimean-Congo hemorrhagic fever virus (CCHFV) is a widely distributed tick-borne member of the *Nairovirus* genus (*Bunyaviridae*) with a high mortality rate in humans. CCHFV induces a severe disease in infected patients that includes, among other symptoms, massive liver necrosis and failure. The interaction between liver cells and CCHFV is therefore important for understanding the pathogenesis of this disease. Here, we described the *in vitro* CCHFV-infection and -replication in the hepatocyte cell line, Huh7, and the induced cellular and molecular response modulation. We found that CCHFV was able to infect and replicate to high titres and to induce a cytopathic effect (CPE). We also observed by flow cytometry and real time quantitative RT-PCR evidence of apoptosis, with the participation of the mitochondrial pathway. On the other hand, we showed that the replication of CCHFV in hepatocytes was able to interfere with the death receptor pathway of apoptosis. Furthermore, we found in CCHFV-infected cells the over-expression of PUMA, Noxa and CHOP suggesting the crosstalk between the ER-stress and mitochondrial apoptosis. By ELISA, we observed an increase of IL-8 in response to viral replication; however apoptosis was shown to be independent from IL-8 secretion. When we compared the induced cellular response between CCHFV and DUGV, a mild or non-pathogenic Nairovirus for humans, we found that the most striking difference was the absence of CPE and apoptosis. Despite the XBP1 splicing and PERK gene expression induced by DUGV, no ER-stress and apoptosis crosstalk was observed. Overall, these results suggest that CCHFV is able to induce ER-stress, activate inflammatory mediators and modulate both mitochondrial and death receptor pathways of apoptosis in hepatocyte cells, which may, in part, explain the role of the liver in the pathogenesis of CCHFV.

## Introduction

Crimean–Congo hemorrhagic fever (CCHF) is a severe tick-born, often fatal, zoonosis caused by Crimean-Congo hemorrhagic fever virus (CCHFV), which is a member of the *Nairovirus* genus within the family *Bunyaviridae* [1]. This family of enveloped viruses is composed of a tripartite, single-stranded RNA negative genome [1]. Its epidemiology reflects the geographical distribution of its vectors (mainly ticks of the *Hyalomma* genus) in more than 30 countries throughout Africa, south-east Europe, the Middle East and Asia [2]–[8]. The geographic range of CCHFV is extensive and it is the second most widespread of all medically important arboviruses after Dengue virus [9]. The mortality rate can be up to 50% in humans and, among other clinical features, severe hemorrhagic manifestations and multiple organ failure are some of the most common symptoms [2], [10]. Damage to endothelial cells and vascular leakage seen in patients may either be a direct result of the virus infection or an immune response-mediated effect [11]. In infected humans, elevated serum levels of acute inflammatory markers such as IL-6, TNF-α, sICAM-1, sVCAM-1, and VEGF-A were correlated to CCHF severity in clinical studies [12], [13] and high levels of IL-8, one of the major mediators of the inflammatory response and a major chemotaxis inducer, were detected in a fatal case of CCHF in Greece [14].

Most of the existing knowledge concerning CCHF pathology originates from autopsies and clinical findings. A retrospective study pointed out the mononuclear phagocytes, endothelial cells and hepatocytes as the main targets of infection [15]. However, the molecular mechanism behind the pathogenesis of CCHF is poorly known. Recently, improvements have been done in the understanding of CCHFV effect on target cells: the replication in antigen presenting cells was demonstrated and the cell response, including the soluble mediators production, was elucidated [16], [17]. Connolly-Andersen et al. described CCHFV's replication and activation of endothelial cells [18]. What is more, two animal models were established to study the CCHFV disease. IFN receptor knockout mice and mice deficient in the STAT-1 signalling were both highly susceptible to CCHFV infection causing rapid onset symptoms, including significant liver damage and death [19], [20], confirming the susceptibility of the virus to interferon host response, that was suggested in *in vitro* studies [21], [22]. The liver appears to be an important target organ for many hemorrhagic fever viruses [23]–[30] including CCHFV. CCHFV is known to feature extensive infection of hepatocytes, with an increase in circulating liver enzymes, swelling and necrosis, however little is known about the involvement of the liver in the outcome of the disease [31].

To better understand the role of the liver in the pathogenesis of CCHFV, we studied the ability of CCHFV to *in vitro* infect and replicate the human hepatocyte Huh7 cell line. We observed the cellular cytopathic effect (CPE) and characterised the molecular mechanisms of the apoptosis induced by CCHFV infection, as well as the cytokine secretion profile of Huh7 cell line. We also analysed the ER-stress profile induced by the CCHFV in this cell line. Then, we used Dugbe virus (DUGV) a mild human pathogen [32] among the closest genetically related Nairoviruses to CCHFV [33], as a model to compare cellular and molecular responses. Our data indicated that these two viruses triggered different responses in this hepatocyte cell line, suggesting that these differences might be relevant for CCHFV pathogenesis understanding.

## Materials and Methods

### Virus preparation and titration

All work with CCHFV was carried out in a BSL-4 and in a BSL-2 for DUGV. CCHFV strain IbAr 10200 and DUGV isolate IbH 11480 (both obtained from Institut Pasteur) were passaged as described elsewhere [16]. Absence of *Mycoplasma* was confirmed using the Mycoalert kit (Lonza, Verviers, Belgium). To produce replication deficient CCHFV, virus stock aliquots were inactivated by UV-light (UV Mineral light lamp, UVG-54, 254 nm, Upland, CA, USA), at a distance of 1 cm on ice for 20 min. The absence of infectivity of the inactivated CCHFV was controlled by infecting 4×10^5^ Vero cells with 250 µl of a pure viral suspension in quadruplicate in a 12 well-plate. No FFU were observed.

Viruses titration was performed as described elsewhere [16].

### Cells and *in vitro* virus infection

Huh7 hepatocarcinoma cell line (CelluloNet, Cat N°120, Lyon, France) was cultured in DMEM (Invitrogen), supplemented with 10% FCS, 5×10^4^ IU Penicillin and 50 mg Streptomycin, 10 mM L-Glutamine, and 0.1 mM of non essential aminoacids (all from Invitrogen). Cells were cultivated at 37°, 5% CO_2_. Absence of *Mycoplasma* was confirmed using the Mycoalert kit (Lonza). Huh7 cells were infected either in a 8-well permanox slide (Nunc, Rochester, NY, USA) or in a 6-well plate (BD) at 7.5×10^4^ and 1.25×10^6^ cells/well respectively. The cells were then infected with CCHFV or DUGV at a MOI of 0.1 and 1, inactivated CCHFV and supernatant from non infected Vero E6 cells (Mock) at 37°C, 5% CO_2_ for 45 min. This moment was considered as time 0, and the course of time started from this point. It should be noted that after the 45 min adsorption period, residual or desorbed virus was eliminated by abundant washing of the cell monolayer. The cells were incubated at 37°C, 5% CO_2_. Cells and supernatants were harvested at 3, 6, 18, 24, 48, 72, 120 and 168 h post infection (p.i.). Cells were harvested in 1 mL of RLT (Qiagen, Courtaboeuf, France) and were stored at −20°C until use. Supernatants were centrifuged at 400 *g* for 5 min, aliquoted and stored at −80°C until use.

### Indirect Immunofluorescence assay

In 8-well permanox slides, CCHFV-infected Huh7 cells were fixed with 3.7% PAF in PBS solution, washed thrice in PBS solution, permeabilised with 0.5% Triton X-100 in PBS solution, and then incubated with primary and secondary specific antibodies as described elsewhere [16]. The cells were examined using an Axio Observer Z.1 (Zeiss, France) and analysed using MetaMorph v7.6 software (Wellcome Trust, UK).

### Quantification of DUGV and CCHFV RNA

Briefly, total RNAs from DUGV- or CCHFV-infected cells were extracted from cell pellets using the RNeasy mini kit (Qiagen) according to the manufacturer's instructions. The S genomes for DUGV and the S genomes and antigenomes for CCHFV were then quantified using a quantitative RT-PCR previously described [16], [34].

### Cytokine detection

Supernatants of Huh7 were analysed to determine the amount of cytokines released using ELISA kits following the manufacturer's protocol. The cytokines tested were IL-1β (Pierce Biotechnology, Rockford, IL, USA), IL-8, IL-6, TNF-α, MIP-1α, MIP-1β, IP-10, RANTES, IL-10 and IL-2 (Bender Medsystem, Vienna, Austria).

### Terminal deoxynucleotidyl transferase-mediated deoxyuridine triphosphate nick end labelling (TUNEL) assay

Huh7 cells, mock-infected and infected with UV-inactivated CCHFV and infective CCHFV at a MOI of 0.1 and 1, were fixed with 3.7% PAF and permeabilised with Triton X-100 (0.1%) in PBS solution. The cells were then washed with PBS solution and subjected to TUNEL assay using an *in situ* cell death detection kit (Roche) according to the manufacturer's instructions. The Epics XL instrument and the Expo32 software (Beckman Coulter) were used and a total of 5 000 events were acquired. The cells were properly gated and a single parameter dot plot of FL2H was recorded.

### Annexin V assay

Huh7 cells were infected, fixed and permeabilised as described in the previous paragraph. The cells were then labelled with FITC-Annexin V, according to the manufacturer's instructions (FITC-Annexin Pharmingen™ Apoptosis Detection Kit I, BD).

### Cell viability determination

Cell viability was determined by the trypan blue exclusion assay. After trypsinisation and washing, viable and unviable CCHFV-infected cells were scored in a Kova Glasstic slide (Hycor Biomedical, Garden Grove, CA, USA) using trypan blue stain 0.4% (v/v). A total of 500 cells per condition were counted.

### Quantitative real-time PCR

The total RNAs were extracted from cell pellets using the RNeasy mini kit (Qiagen) following the manufacturer's protocol and were reverse transcribed using the Improm II kit (Promega). Quantitative real-time PCR commercial kits (Search-LC, Roche, Heidelberg, Germany) were used to quantify the expression of genes coding for cytokines: TNF-α, IL-8 and IL-6; apoptosis pathways proteins: BAX, Bcl-2, Bcl-xL and the housekeeping gene PBGD. HRK, PERK, CHOP, PUMA and Noxa were quantified following the real-time PCR protocols described by others, respectively [35]–[37]. The levels of RNA were normalised according to the PBGD mRNA level, which was amplified in duplicate for each of the tested mRNAs using a Bio-Rad CFX96 (Bio-Rad, Hercules, CA, USA). For each mRNA, the ratio value was obtained as follows: ratio of mRNA of interest = 2^−[(Ct gene of interest – Ct PBGD) infected – (Ct gene of interest – Ct PBGD) mock]^.

### Detection of messenger RNA

Semi-quantitative conventional PCR tests were used to detect XBP1 [38] and TRAIL [39]. The expected sizes of PCR-amplified fragments were 416 bp for XBP1 (if any alternative splicing is observed) and 442 bp (for the unspliced form) 494 bp for TRAIL (if any alternative splicing is observed) and 537 bp (for the unspliced form). The expected amplicon size for PBGD was 341 bp. Cycling conditions were those described by Klomporn *et al.*, 2011 [40]. Accurate separation and sizing of spliced variants of TRAIL and XBP1 was done using the Agilent DNA 1000 Chip (Agilent Technologies, Waldbronn, Germany).

### Cloning and sequencing XBP1 and TRAIL splice variants

Amplicons of TRAIL, XBP1 and the correspondent splice variants were obtained by RT-PCR as described above, ligated into the pCR2.1-TOPO vector (Invitrogen) and cloned in accordance to standard protocols. Clones were sequenced by GATC Biotech (Mulhouse, France) using T7 and M13R site-specific primers.

### IL-8 neutralization assay

Confluent Huh7 cells were pre-incubated with or without neutralising antibody against IL-8 (50 µg/ml; catalog no. AB-208-NA; R&D Systems, Lille, France) diluted in fresh medium, for 3 h at 37°C, 5% CO_2_. CCHFV infection was performed as described in the cells and *in vitro* virus infection paragraph. After infection the same suspension of IL-8 was added to the infected cells. IL-8 was quantified using the ELISA kit previously described, to control its neutralisation.

### Statistical analysis

The Student t test was used to compare two sets of data with a P value<0.05 considered significant. Standard deviation (SD) were determined using the Excel® SD function (Microsoft).

## Results

### CCHFV and DUGV infect and replicate in Huh7 cell line

**Figure 1:**
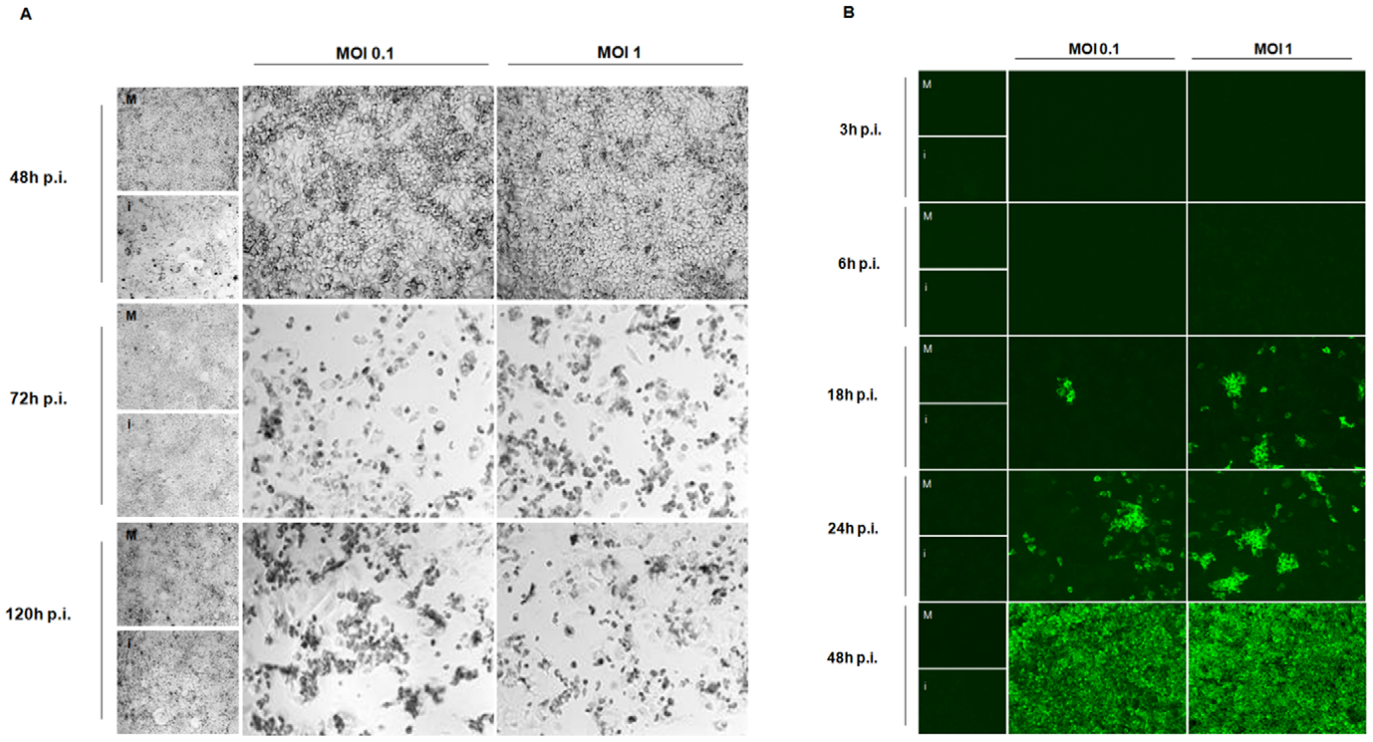
CCHFV effects and expression in Huh7 cell line. (A) Optical photomicrography of CCHFV-infected Huh7 monolayers at MOIs 0.1 and 1. Observations were performed from 48 to 120 h p.i.: on the left are represented mock-infected cells (M) and UV-inactivated CCHFV-infected cells (i); on the center CCHFV-infected Huh7 at a MOI of 0.1; on the right CCHFV-infected Huh7 at a MOI of 1. Data represents one out of three experiments performed in duplicate. Magnification: 20×. (B) Fluorescent photomicrography of CCHFV-infected Huh7 monolayers (MOIs 0.1 and 1), incubated with a specific anti-CCHFV polyclonal antibody. Observations were performed from 3 to 48 h p.i.: on the left are represented mock-infected cells (M) and UV-inactivated CCHFV-infected cells (i); on the center CCHFV-infected Huh7 at a MOI of 0.1; on the right CCHFV-infected Huh7 at a MOI of 1. Data represents one out of three experiments performed in duplicate. Magnification: 20×.

**Figure 2:**
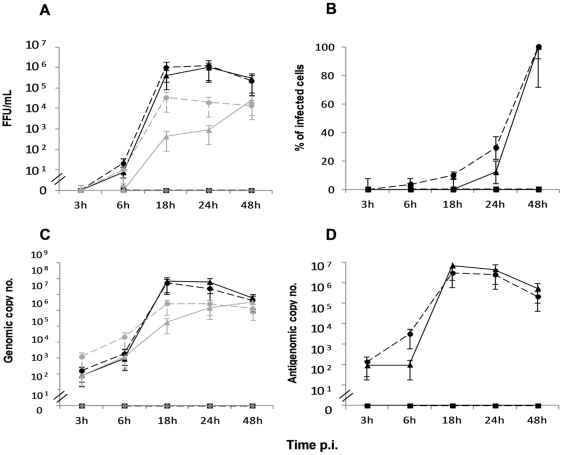
Sensitivity and permissivity to CCHFV and DUGV. (A) CCHFV- and DUGV-infected cells were assayed for the cell supernatant titres, using a specific polyclonal antibody, expressed in FFU/ml; (B) the percentage of CCHFV-infected cells, calculated using the fluorescent photomicrography and analysed using Metamorph v7.5; (C) the genomic strand was assayed for CCHFV and DUGV-infected Huh7 cells by real time qRT-PCR. (D) the antigenomic strand copy number from cellular extracts of CCHFV-infected cells, obtained by real time qRT-PCR. Means ± sd three independent experiments performed in duplicate are represented. ―♦― mock-infected cells; --•--, infection at MOI of 1; ―▴―, infection at MOI of 0.1 (CCHFV in black and DUGV in grey); --▪--, UV-inactivated CCHFV.

During human infection with CCHFV, hepatocytes are considered to be one of the main target cells [15]. To further test the susceptibility and the response of these cells to CCHFV infection, we used in our *in vitro* model experiments, the Huh7 cell line derived from hepatocellular carcinoma. Each experiment presented in this work represents the results obtained from three independent experiments, performed in duplicate. Daily, CCHFV-infected Huh7 cells were examined microscopically for the appearance of CPE compared to mock-infected cells and UV-inactivated CCHFV-infected cells. Marked CPE and cell death were observed from 72 through 120 h p.i. (figure 1; Panel A) leading us to define 48 h p.i., as the maximal time point of the kinetics for all experiments. Viral antigens determined by indirect immunofluorescence assay (IFA) (figure 1; Panel B), were detected from 18 through 48 h p.i.. It appears that the ability of CCHFV to infect Huh7 was similar for both MOIs (0.1 and 1) but slightly higher for MOI 1 at 18 h and 24 h p.i. (5.1% versus 9.8%, p<0.05 at 18 h p.i. and 12.1% versus 29.2%, p<0.05 at 24 h p.i. for MOI 0.1 and 1, respectively). It is important to point out that at 48 h p.i., 100% of the cell monolayer was found positive by IFA (figure 1; Panel B and figure 2; Panel B). UV-inactivated CCHFV-infected cells did not exhibit detectable viral antigens. The replicative virus released into the medium was detected as soon as 6 h p.i. for both MOIs. The viral growth curve (figure 2; Panel A) indicated that the viral production peaked at 18 h p.i. (1.0×10^6^ FFU/mL), smoothly decreasing 1 log_10_ from 24 to 48 h p.i. (2.3×10^5^ FFU/mL). When cells were infected at the lowest MOI, the viral yield was similar to that of MOI 1 except for 18 h p.i. where the titre decreased slightly (4.2×10^5^ FFU/mL). Accordingly, the kinetics of viral production had the same profile. In the experiments performed at times higher than 48 h p.i., a decrease of the titre was observed, for example, a titre of 1.6×10^4^ FFU/mL was obtained at day 3 p.i., for MOI 1 (data not shown). UV-inactivated CCHFV-infected cells did not display any foci.

To further demonstrate CCHFV replication, we assayed a quantitative strand specific RT-PCR detecting either the genomic or the antigenomic strand of the S segment. Detection of the genomic and antigenomic strand was performed using total RNA extracted from the infected cells. The kinetic curve of the genomic strand copies (figure 2; Panel C) showed that CCHFV genome began to replicate as soon as 3 h p.i., it increased abruptly until 18 h p.i., plateaued from 18 to 24 h p.i. and decreased steadily until 48 h p.i. We also assayed the antigenomic S RNA, synthesized in infected cells (figure 2; Panel D). As expected, in the mock and UV-inactivated controls the antigenomic strand was not detected. High amounts were detected as early as 3 h p.i. in CCHFV-infected Huh7 cell line at both MOIs (9.1×10^1^ and 1.4×10^2^ copies/mL for MOI 0.1 and 1, respectively). The number of antigenome molecules declined slightly at 48 h p.i (figure 2; Panel D). Both genomic and antigenomic RNA strand profiles were correlated with the profile of the replicative particles with a ratio 1 to 20 genomic strand for 1 antigenomic replicative intermediate.

Like CCHFV, DUGV was found to replicate in these cells (figure 2; Panel A and C), however, we found that DUGV titres and genomic copy numbers were from 10 to 1000 times lower than CCHFV when infected at the highest MOI. For MOI 0.1 the difference was more pronounced. The DUGV titres and genomic copy numbers curve had a similar profile peaking at 18 h p.i.. Moreover, DUGV replication profile for MOI 1 was similar to CCHFV. Despite the virus replication, no CPE were observed in Huh7 cells at any time of the kinetic (data not shown).

### CCHFV and DUGV increase the secretion of IL-8 in Huh7 cell lines

**Figure 3:**
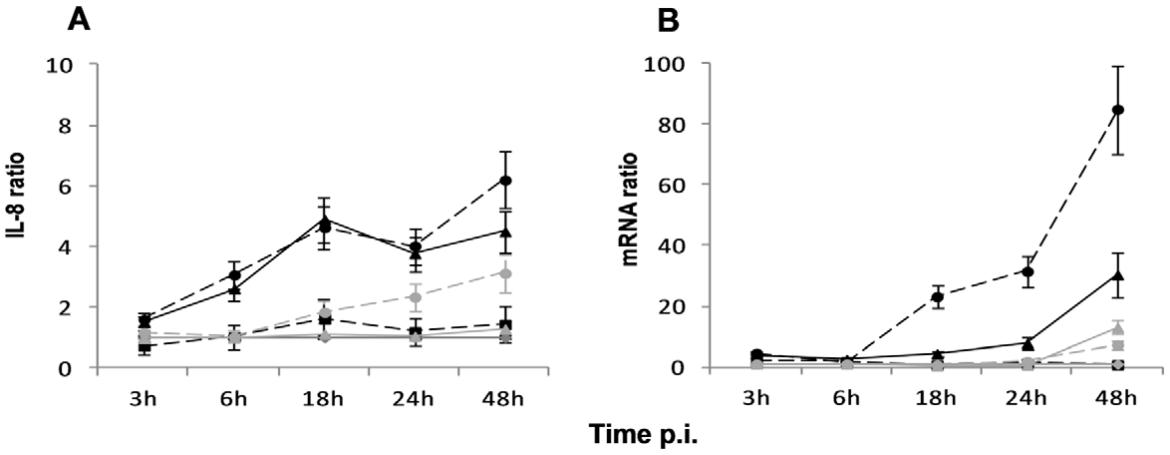
IL-8 release induced by CCHFV is higher than that induced by DUGV. (A) IL-8 released into the maintenance medium was assayed by ELISA and represented as IL-8 fold increase ratio comparing to mock-infected cells; (B) Expression levels of the IL-8 gene were quantified by by real time qRT-PCR, using mean ± sd of three independent experiments performed in duplicate. ―♦― mock-infected cells; --•--, infection at MOI of 1; ―▴―, infection at MOI of 0.1 (CCHFV in black and DUGV in grey); --▪--, UV-inactivated CCHFV.

Following the infection of Huh7 cell line with CCHFV, the release of several mediators with a potential role in the pathogenic cascade was tested. Among all soluble mediators potentially secreted by hepatocytes tested in our experiments including IL-1β, IL-8, IL-6, TNF-α, MIP-1α, MIP-1β, IP-10, RANTES, IL-10 and IL-2, IL-8 was the only one to be modulated by CCHFV from 18 to 48 h p.i. (figure 3; Panel A). The over-secretion of IL-8 started between 3 and 6 h p.i. and increased until 48 h p.i., where it reached its maximum 4.6-fold (p<0.05) at the highest MOI (MOI 1) when compared with the mock-infected cells. UV-inactivated CCHFV-infected cells elicited an increase of 1.6-fold when compared to mock-infected cells. We also observed the up-regulation of IL-8 mRNA (figure 3; Panel B). Moreover, we confirmed by quantitative RT-PCR, the absence of the soluble mediators including TNF-α and IL-6, by testing the corresponding cytokines' genes (data not shown).

When we investigated the response triggered by DUGV in Huh7 cells, we observed that like CCHFV, DUGV over-expressed only IL-8 (3.1-fold (p<0.05) at 48 h p.i.) (figure 3 and Table 1). This modulation was 1.5 times lower than the one observed for CCHFV at 48 h p.i..

**Table 1:** mRNA modulation of CCHFV-infected versus DUGV-infected Huh7 at different times post infection.

	CCHFV			DUGV		
	18 h	24 h	48 h	18 h	24 h	48 h
Inflammatory response genes						
IL-8	+*	+*	+*	−	−	+
Mitochondrial apoptotic pathway genes						
BAX	+*	−	−	−	−	−
HRK	+	+*	+*	+	+	+
Bcl-xL	−	+	−	−	−	−
Bcl-2	−	−	−	−	−	−
ER-stress genes						
XBP1s	+*	+	+	+	∼	∼
PERK	+	+	−	+	+	−
ER-stress/apoptosis crosstalk genes						
PUMA	+	+	+	−	−	−
Noxa	−	−	∼	−	−	−
CHOP	+	+	+	−	−	−
Death receptor apoptotic pathway genes						
TRAIL-R2	−	−	−	−	−	−
TRAILs	+	+	+	−	−	−

*significantly higher for CCHFV-infected Huh7 cells.

+ positive.

− negative.

∼ slightly positive.

### Huh7 cell death at 72 h p.i. is due to CCHFV-induced apoptosis

**Figure 4:**
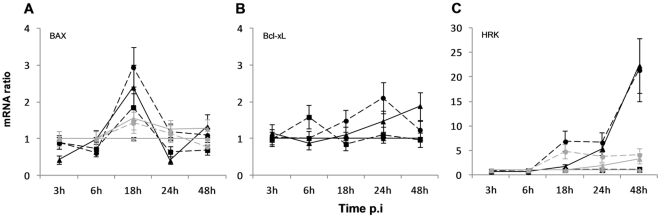
CCHFV modulates members of the Bcl-2 family of genes. Expression levels of (A) BAX, (B) Bcl-xL and (C) HRK genes were quantified by real time qRT-PCR, using mean ± sd of two independent experiments performed in duplicate. ―♦― mock-infected cells; --•--, infection at MOI of 1; ―▴―, infection at MOI of 0.1 (CCHFV in black and DUGV in grey); --▪--, UV-inactivated CCHFV.

To determine whether the CPE observed for CCHFV-infected Huh7 cell line at 72 h p.i. was due to apoptosis, a TUNEL assay was performed at 3, 24 and 48 h p.i., as endonucleolysis is considered one of the key biochemical events of apoptosis. The number of CCHFV-infected TUNEL-positive cells for MOI 1 was 13.5% at 48 h p.i. (p<0.05), compared to 0.16% for the mock-infected cells and 0.23% for the UV-inactivated CCHFV-infected cells. Lower amounts of oligonucleosomal length DNA were detected at 24 h p.i.: 2.2% for CCHFV-infected Huh7 (p<0.05), compared to 0.64% for the mock-infected cells and 0.23% for the UV-inactivated CCHFV-infected cells (data not shown). Consistent with the CPE effects, no major difference was observed between MOI 0.1 and MOI 1. Annexin V experiments confirmed the results obtained with the TUNEL assay (data not shown). To further confirm the apoptotic effect observed in CCHFV-infected Huh7 cells, the up-regulation of the expression the Bcl-2 family of genes was investigated due to its role in the regulation of programmed cell death. The results obtained are consistent with those described for the TUNEL assay, i.e. a significant increase in the expression of BAX and HRK genes was observed; and only a slight increase of Bcl-xL gene and no expression modulation of Bcl-2 gene were detected (figure 4; Panel A–C). The up-regulation of BAX mRNA expression in CCHFV-infected cells relative to mock infected cells was only observed at 18 h p.i. with a ratio of 2.9-fold increase (p<0.05) for MOI 1 (figure 4; Panel A). For MOI 0.1 and UV-inactivated CCHFV-infected cells the increase was not statistically significant. Moreover, HRK gene expression was significantly up-regulated for both MOIs, reaching 22-fold (p<0.01) over-expression at 48 h p.i. (figure 4; Panel C) when compared to mock-infected cells and UV-inactivated CCHFV-infected cells. Bcl-xL displayed an up-regulation of 2.1-fold at 24 h p.i. for MOI 1 and 1.9-fold at 48 h p.i. for MOI 0.1 (figure 4; Panel B). Furthermore, the ratio of BAX/Bcl-2 genes, which appears to determine whether some cells live or die, was found to be positive, corresponding to the up-regulation of the expression of BAX gene. Cell viability, characterised by trypan blue exclusion test cell counts from 3 to 48 h p.i., was determined. We observed at 48 h p.i., 4.5, 6.0 and 7.3% of dead cells for the mock-infected and CCHFV-infected cells at MOI 0.1 and 1, respectively.

In DUGV-infected cells, no TUNEL-positive cells were found from 3 to 48 h p.i. However, BAX and HRK genes were over-expressed in significant lower amounts when compared to CCHFV (figure 4 and Table 1).

### CCHFV and DUGV infection trigger ER-stress in Huh7 cells

**Figure 5:**
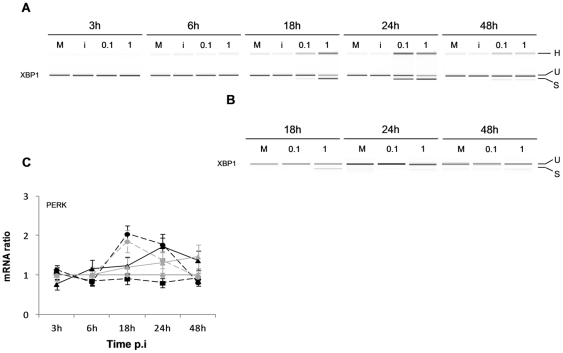
CCHFV and DUGV induce ER-stress. CCHFV-infected cells were examined for the (A) IRE1-mediated XBP-1 splicing by conventional RT-PCR (H is the heteroduplex, U the unspliced and S the spliced form of the XBP1 transcript) or (B) the expression levels of PERK by real time qRT-PCR, using mean ± sd of two independent experiments performed in duplicate. ―♦― mock-infected cells; --•--, infection at MOI of 1; ―▴―, infection at MOI of 0.1 (CCHFV in black and DUGV in grey); --▪--, UV-inactivated CCHFV.

We hypothesized that CCHFV triggered ER-stress in Huh7 cells. We examined the induction of two ER-stress pathways, the Unfolded Protein Response (UPR) and Noxa/PUMA in response to CCHFV infection in Huh7 cells from 3 to 48 h p.i.. For the UPR, we investigated the IRE1 mediated splicing of the XBP1 transcript, a hallmark of UPR induction, and the up-regulation of the expression of PERK, one of the transmembrane sensors of ER-stress, together with IRE1 [41]. Results demonstrated a clear induction of the alternate splicing of XBP1 at 18 h p.i. with a peak at 24 h p.i. for both MOIs (figure 5; Panel A). These results were confirmed by XBP1 sequencing (data not shown). Of note, the sequence analysis showed that the spliced variant gene corresponded to the variant 2 of XBP1 (accession number NM_001079539). Mock-infected cells as well as UV-inactivated CCHFV-infected cells did not display any visible splicing of XBP1. As noted by others, we also observed the presence of an additional band (named H, figure 5; Panel A), which is believed to be a heteroduplex product between the spliced and the unspliced products [40], [42], [43]. An up-regulation of the expression of PERK gene was also observed at 18 h and 24 h p.i. for MOI 1 and MOI 0.1 respectively, and decreased after these time points. Figure 5; Panel C illustrates that at 18 h p.i. the expression in CCHFV-infected Huh7 cells was double comparing to mock-infected cells and UV-inactivated CCHFV-infected cells.

### CCHFV-infected hepatocytes induce ER-stress and apoptosis crosstalk, but not DUGV

**Figure 6:**
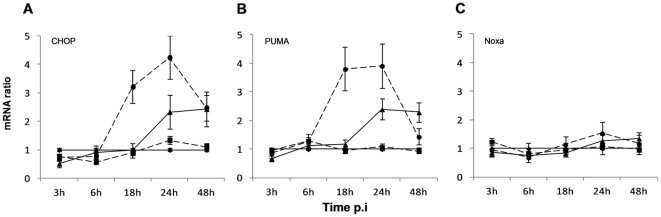
CCHFV upregulates the expression of ER-stress-induced apoptotic genes. Expression levels of (A) CHOP, (B) PUMA and (C) Noxa genes were quantified by real time qRT-PCR, using mean ± sd of two independent experiments performed in duplicate. ―♦― mock-infected cells; --•--, infection at MOI of 1; ―▴―, infection at MOI of 0.1 (CCHFV in black and DUGV in grey); --▪--, UV-inactivated CCHFV.

In search of the molecular link between ER-stress and the mitochondria, we also examined the expression of CHOP mRNA, as it is one of the apoptotic proteins up-regulated by the UPR. The expression of CHOP mRNA increased from 6 h p.i. and reached 4.2-fold at 24 h p.i. when MOI 1 was used; at an MOI 0.1 it increased from 18 h p.i. and reached 2.5-fold at 48 h p.i. when compared to mock-infected cells (p<0.05) (figure 6; Panel A). In addition to the UPR, the Noxa/PUMA ER-stress pathway was also investigated. The mRNA expression of the genes PUMA and Noxa in Huh7 cells was analysed. As shown in figure 6; Panel B, an increase of PUMA mRNA expression was observed in CCHFV-infected cells from 6 h p.i., peaking at 24 h p.i. with a fold increase of 3.9 when MOI 1 was used; and starting from 18 h p.i. when MOI 0.1 was used, with a peak of 2.4-fold at 24 h p.i. related to mock-infected cells (p<0.05). A slight up-regulation of Noxa mRNA was detected by 48 h p.i. for MOI 0.1, when compared to mock-infected cells and UV-inactivated CCHFV-infected cells (figure 6; Panel C). The increase observed by 24 h p.i. at MOI 1 was statistically not significant (p>0.05).

Lower levels of XBP1 splicing were observed in the DUGV model (figure 5; Panel B). The up-regulation of the PERK gene expression was comparable to that obtained for CCHFV (figure 5; Panel C and Table 1). No up-regulation of CHOP, PUMA and Noxa was observed in DUGV-infected Huh7 (data not shown).

### CCHFV infection induces splicing of TRAIL mRNA in Huh7 cells

**Figure 7:**
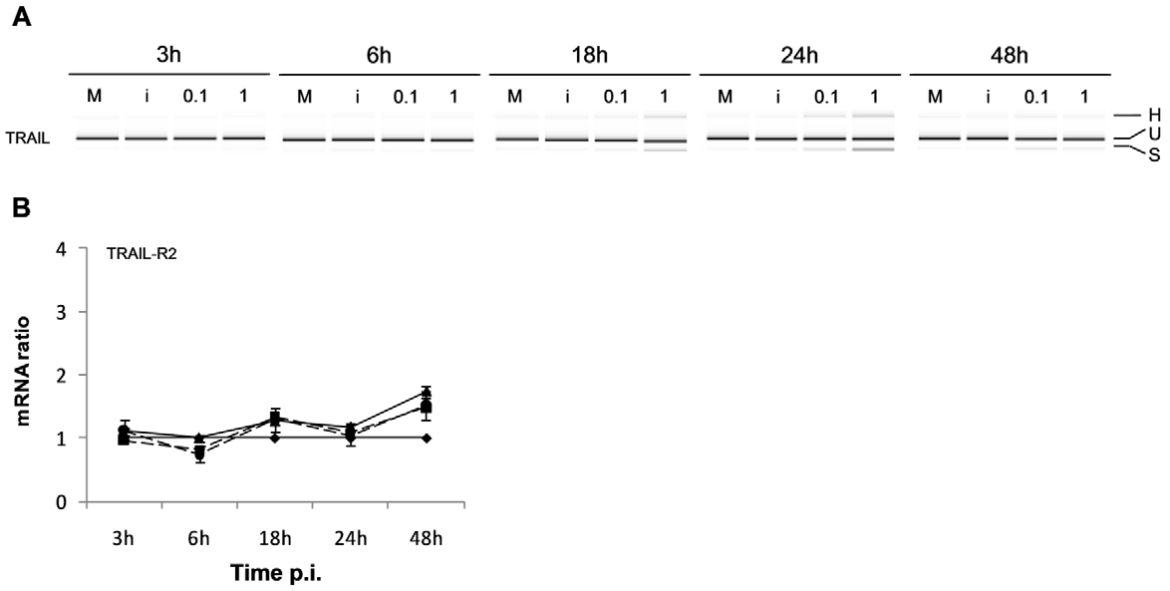
CCHFV induces TRAIL alternative splicing. CCHFV-infected cells were examined for the (A) the expression levels of TRAIL by conventional RT-PCR (H is the heteroduplex, U the unspliced and S the spliced form of the TRAIL transcript) or (B) the expression levels of TRAIL-R2 by real time qRT-PCR, using mean ± sd of two independent experiments performed in duplicate. --•--, CCHFV at MOI of 1; ―▴―, CCHFV at MOI of 0.1; --▪--, UV-inactivated CCHFV; ―♦― mock-infected cells.

To determine if the apoptotic features observed in CCHFV-infected Huh7 cells derives only from the intrinsic pathway or to a coordination between the intrinsic and the extrinsic pathways, the mRNA levels of the death receptor TRAIL-R2 and its ligand TRAIL were assessed. The results obtained showed that neither TRAIL-R2 nor TRAIL were significantly expressed (figure 7; Panel A–B). We observed, in CCHFV-infected cells (18, 24 and 48 h p.i.), that two additional bands co-amplified with the expected TRAIL amplicon, one of 43 bp shorter and another of 160 bp longer than the expected amplicon (figure 7; Panel A). Sequence analyses showed the presence of a splicing variant of TRAIL (TRAIL-ß) described elsewhere [44]. Interestingly, the splicing site of TRAIL-ß mRNA was located one nucleotide downstream of the splicing site already described. The upper additional band, which could correspond to a heteroduplex, was not sequenced due to its very low concentration. When we examined the expression of TRAIL, we observed that, in contrast with CCHFV, DUGV did not induce any expression of TRAIL-β (data not shown).

### CCHFV replication and apoptosis induction is not IL-8-dependent

IL-8 has been described to have both apoptotic [45] and anti-apoptotic [46] functions. Furthermore, IL-8 protein levels were described to be positively associated with virus replication *in vitro* [47]. To investigate the relationship between IL-8 over-expression, virus replication and apoptosis induction, IL-8 protein was neutralised during the CCHFV kinetic by adding anti-IL-8 polyclonal antibodies until the protein levels of IL-8 were undetectable by ELISA. We found that neither the CCHFV replication nor the mRNA levels of apoptotic genes were modified after IL-8 neutralisation (data not shown).

## Discussion

The hepatocytes, together with the mononuclear phagocytes and the endothelial cells, are considered target cells of CCHFV infection in humans [15]. Little is known about the pathogenesis mechanisms of CCHFV. So far, only few *in vitro* studies have focused on the effects of CCHF on mononuclear phagocytes [16], [17], endothelial cells [18] and hepatocyte cells [21], [22]. That is why we extensively studied the *in vitro* interaction between CCHFV and hepatocytes in order to correlate these interactions with CCHF pathogenesis. Our main findings were that CCHFV induced ER-stress, activated inflammatory mediator and modulated both intrinsic and extrinsic pathways of apoptosis in hepatocytes, which are key target cells. The comparison with a non-pathogenic nairovirus response highlighted the contribution of the ER-stress and apoptosis crosstalk in CCHFV-infected liver cells. These molecular and cellular mechanisms observed *in vitro* could be relevant factors which could explain why CCHFV is extremely pathogenic.

According to our results, CCHFV infection was considered to be rapid and to replicate to high loads in Huh7 cell line, when compared to the replication in other *in vitro* CCHFV-infected human cells, including moDCs, moMPs, HUVEC, A549 and SW13 cells [16]–[18], [21], [22]. When compared to DUGV infection in Huh7 cells, CCHFV was shown to replicate better in Huh7 cells. Furthermore, the CCHFV titres obtained for Huh7 cells were in accordance with those obtained by others [21], [22]. These features suggest that the liver may play an important role in the amplification of CCHFV load, which is a key element in the pathogenesis of the disease and is considered as a predictor of mortality [48]. The ability of CCHFV to replicate in liver cells was also proven *in vivo* in mice experiments: liver was shown to display the highest levels of CCHFV load. [19], [20], .We further observed that CCHFV at a MOI 0.1 rapidly attained the same rate of replication as CCHFV at a MOI 1 and that the percentage of infected cells, reaching 100%, was similar for both concentrations of the virus throughout the 48 h of infection. A comparable result was observed in *in vivo* experiments, for which, independently of the dose of virus used for the challenge, similar titres of viral RNA were found in the organs [19]. At 72 h p.i., when the strong CPE occurred at both MOIs, most of the CCHFV-infected cells of the monolayer were detached when compared to mock-infected cells. The absence of CPE in UV-inactivated CCHFV-infected cells suggests a direct effect of CCHFV replication. Comparing to the lack of CPEs during DUGV infection, we suggested that CPEs could be an *in vitro* marker of CCHFV pathogenicity. Since the CPEs were observed after 72 h p.i., when CCHFV replication had already reached its maximum at 18 h p.i., we are tempted to speculate that CCHFV-induced CPE was a late event, allowing efficient viral production and spread. These results leaded us to suggest that in humans, after a tick bite, and after the APCs provided systemic dissemination of the virus [16], [17], [49], CCHFV reaches the liver, which might be one of the main sites of CCHFV replication, before or simultaneously disseminating systemically in greater amounts into multiple organs.

To determine the cause of the CPE, we tested two different morphologic markers of apoptosis. The ability of bunyaviruses to induce apoptosis *in vitro* was already described, including in CCHFV-infected SW13 cell line [50]–[55]. The detection of apoptosis before the CPE in CCHFV- but not in DUGV-infected Huh7, suggests that CCHFV-induced CPE could be the consequence of apoptosis in Huh7 cells. Furthermore, we found that the capacity of CCHFV to elicit apoptosis in hepatocytes was dependent on the ability of the virus to replicate, given that UV-treated virus failed to cause apoptosis. This indicates that viral replication-associated products may be essential to trigger apoptosis.

In order to characterise the molecular mechanisms that elicited CCHFV-induced apoptosis, we investigated the differential expression of several proteins of the Bcl-2 family at the mRNA level. The Bcl-2 family of proteins has been shown to be the central regulator of the intrinsic apoptotic pathway [56]. Bcl-xL and Bcl-2, two anti-apoptotic members, have been found to block apoptotic cell death by, respectively, interaction and heterodimerization with BAX, which is a potent mediator of programmed cell death.. We demonstrated a differential regulation of endogenous BAX mRNA but not Bcl-2 gene and only a minor up-regulation of Bcl-xL expression in response to the CCHFV replication, obtaining a positive BAX/Bcl-2 ratio comparing to mock-infected cells and UV-inactivated CCHFV-infected cells. It is important to note that the increase of BAX mRNA at 18 h p.i. was concomitant with the peak of viral production, suggesting a possible link between the up-regulation of BAX and CCHFV replication. The absence of differential expression of Bcl-2 was not surprising since several authors described the absence of Bcl-2 expression in hepatocytes [57], [58]. The ratio BAX/Bcl-xL was also positive at 18 h p.i., corresponding to the peak of up-regulation of BAX. These results strongly suggest that CCHFV-induced apoptosis involves the mitochondrial pathway. To further confirm the involvement of the mitochondrial pathway, the up-regulation of the expression of the BH3-only genes of the Bcl-2 family HRK, PUMA and Noxa was investigated. These proteins are essential initiators of apoptotic cell death [59] through their interaction with the anti-apoptotic members of the Bcl-2 family [59], [60]. We clearly showed a strong up-regulation of the expression of the HRK gene in CCHFV-infected cells comparing to mock-infected and UV-inactivated CCHFV-infected cells (figure 6). Both anti-apoptotic proteins (Bcl-xL and Bcl-2) have been found to interact with HRK, inhibiting its death-promoting activity [61]. Similarly to BAX, the balance between the levels of Bcl-xL and Bcl-2 and those of HRK appears to modulate apoptosis. Thus, the strong up-regulation of HRK but not Bcl-2 as well as the minor Bcl-xL up-regulation provides evidence that the viral infection was able to trigger apoptosis. Taken together, we illustrated in our experiments CCHFV-induced apoptosis in Huh7 with the participation of the mitochondrial pathway. Many studies have shown that viruses are able to modulate mitochondrial apoptosis (reviewed by Galluzzi et al, 2008) [62]. For example, Dengue virus, which can also induce a hemorrhagic fever, was described to decrease the expression of Bcl-2 and Bcl-xL in both mRNA and protein levels [63]. Vesicular Stomatitis Virus was shown to decrease the cellular levels of Bcl-xL while maintaining the levels of BAX [64]. The envelope glycoprotein complex encoded by HIV-1 was described to induce the expression of BAX [65]. HCV was proven to induce apoptosis through the mitochondrial pathway by down-regulating Bcl-2 and up-regulating BAX protein expression [66].

The increase in the transcription of PUMA in response to CCHFV infection not only emphasizes the participation of the intrinsic pathway, but also suggests the crosstalk between the ER and the mitochondrial apoptosis machinery, specifically through the Noxa/PUMA pathway. These BH3-only genes of the Bcl-2 family were recently described as novel components of the ER-stress-induced apoptotic pathways [67]. Under conditions of ER-stress, the transcriptional activation of Noxa and PUMA by p53 leads to the induction of apoptosis by the release of cytochrome C from the mitochondria as a result of the activation of Bak or BAX [67]. In CCHFV-induced apoptosis the over-expression of PUMA at the mRNA level was greater and was observed earlier than Noxa, suggesting a more important role for PUMA. The over-expression of CHOP gene mRNA, a transcription factor that can be activated in cells suffering from ER-tress during the UPR [68], was also observed in CCHFV-infected Huh7 cells. In CCHFV-infected Huh7 cells, CHOP differential expression appeared to match well with the CCHFV-replication.. As documented here, an increase in the transcription of CHOP and PUMA occur in response to CCHFV replication (figure 6), confirming the involvement of the mitochondrial pathway in CCHFV-induced apoptosis and demonstrating the activation of multiple pathways of crosstalk between the ER and the mitochondrial apoptosis machinery. Furthermore the up-regulation at the mRNA level of the apoptotic genes BAX and HRK was found to be lower in DUGV-infected comparing to CCHFV-infected Huh7 cells (figure 4), and the expression of CHOP and PUMA mRNA was not observed in DUGV-infected Huh7 cells during the kinetic. This reinforces the possible specificity of apoptosis to CCHFV infection in Huh7 cells. We hypothesized that CCHFV-induced ER-stress, could possibly be the cause of apoptosis in hepatocytes cells. We assessed the alternative splicing of the XBP1 gene, a hallmark of ER-stress and the differential expression of the gene coding for PERK, one of the three key sensors of ER-stress present on the ER membrane [41]. The presence of the spliced variant of XBP1 and the up-regulation of the PERK gene (figure 5) were concomitant with CCHFV replication. Furthermore, DUGV also induced ER-stress, comparable to CCHFV but in a lower manner. This suggests that despite a similar ER-stress profile, the ER-stress response acts as a survival mechanism in DUGV-infected cells.

Since the ER serves as the primary site of replication for many enveloped viruses [5], viral infections associated with synthesis of large amounts of viral proteins in a short amount of time perturbs ER homeostasis [69], [70]. Moreover, it has been shown that over-expression of viral components alone in cultured cells is enough to induce ER-stress mediated apoptosis [71], [72]. Thus we suggest that the demand of synthesis and folding of high amounts of CCHFV proteins in such a short period of time leads to ER-stress in CCHFV-infected Huh7 cells. We showed here that both known ER-stress pathways are triggered after CCHFV infection: the UPR and the Noxa/PUMA pathway. We also showed that apoptosis is mediated by the mitochondrial pathway and that there is a crosstalk between the ER and the mitochondrial apoptosis. Thus, it is tempting to assume that CCHFV-induced mitochondrial apoptosis is ER-stress mediated, although studies blocking the ER-stress pathways are needed to further prove this assertion.

Apart from the cellular perturbations that comprise the intrinsic pathway of apoptosis, hepatocyte apoptosis can also be initiated via the death receptor or extrinsic pathway of apoptosis [73]. Since the secretion of TNF-α was not observed in CCHFV-infected Huh7 cells, we investigated the modulation of the expression of TRAIL and its receptor TRAIL-R2. Interestingly, despite the absence of a differential expression of TRAIL-R2 or TRAIL genes, the detection of a spliced variant of TRAIL, only in CCHFV-infected Huh7 cells (figure 7) suggested a modulation of this pathway. This spliced variant, proven to correspond to TRAIL-β, undergoes an extensive loss of its extracellular binding domain, being unable to form stable ligand-receptor complexes and failing to trigger TRAIL-mediated apoptosis [44]. This result provides evidence that CCHFV may interfere with the fine tuning of TRAIL simply by reducing the amount of full length ligand, and it was emphasised by the absence of splicing during DUGV-infection. However, further studies are needed to understand the biological significance of this interesting feature.

In our *in vitro* model, we also observed an increase of IL-8 transcription and secretion into the supernatants of CCHFV-infected Huh7 cells, which were shown to be higher than in the supernatants of DUGV-infected cells. The increase of IL-8 could be a consequence of CCHFV-induced cellular stress and activation of the inflammatory response. Indeed, it has been shown that IL-8 can be induced in response to cellular stress, including Hepatitis C virus [28] or Dengue virus [74] infection. It is known that IL-8 increases endothelial cell permeability [75]–[77], suggesting a role for IL-8 in plasma leakage, which is a hallmark of CCHF. Recently Connolly-Andersen *et al.* described the secretion of IL-8 in CCHFV-infected endothelial cells [18] Thus, it could be interesting to test IL-8 in infected patients to assess the potential of IL-8 as a marker of the disease severity. IL-8 has also been described to enhance endothelial cell proliferation and survival by the expression of anti-apoptotic genes [45] but one study correlated IL-8 secretion with the induction of apoptosis in leukemic cells [46]. To determine the possible link between IL-8 and CCHFV replication and/or CCHFV-induced apoptosis, a commercial neutralizing antibody directed against IL-8 was used. In our experiments, CCHFV replication and CCHFV-induced apoptosis were independent from CCHFV-induced IL-8 up-regulation.

Altogether, these results showing differences of cellular response between these two nairoviruses with such a different clinical outcome, temptingly, offer elements to explain the pathogenesis of the CCHFV.

In summary, we think that our findings could provide new approaches to understand the molecular and cellular mechanisms of pathogenesis of CCHFV infection.
